# Supplementary material for: Modeling of magnetic vestibular stimulation experienced during high-field clinical MRI
Source: Commun Med (Lond). 2025 Jan 21;5:27. doi: 10.1038/s43856-024-00667-9 (PMC11751175; doi:10.1038/s43856-024-00667-9)
Supplement: Supplementary file 2 — Supplementary Information [file 43856_2024_667_MOESM2_ESM.pdf]

SUPPLEMENTARY INFORMATION

Modeling of magnetic vestibular stimulation experienced during high-field clinical MRI

Ismael Arán-Tapia[123], Vicente Pérez-Muñuzuri[13], Alberto P. Muñuzuri[12], Andrés Soto-Varela[456], Jorge Otero-Millan[7], Dale C. Roberts[8] & Bryan K. Ward[9]

[1] Group of Non-Linear Physics, University of Santiago de Compostela, Santiago de Compostela, Spain.

[2] Galician Center for Mathematical Research and Technology (CITMAga), Santiago de Compostela, Spain.

[3] CRETUS Institute, Santiago de Compostela, Spain.

[4] Division of Neurotology, Department of Otorhinolaryngology, Complexo Hospitalario Universitario, Santiago de Compostela, Spain.

[5] Department of Surgery and Medical-Surgical Specialities, Universidade de Santiago de Compostela, Spain.

[6] Health Research Institute of Santiago (IDIS), Santiago de Compostela, Spain.

[7] The University of California, Berkeley, School of Optometry, Berkeley, California, USA.

[8] Department of Neurology, Johns Hopkins University School of Medicine, Baltimore, Maryland, USA.

[9] Department of Otolaryngology-Head and Neck Surgery, Johns Hopkins University School of Medicine, Baltimore, Maryland, USA.

Supplementary Method 1
Boundary conditions

To effectively solve the Electrodynamic Potential model within the context of our study, appropriate boundary conditions had to be established for the dark and hair cell regions in the membranous labyrinth. The model treated the other walls of the membranous labyrinth as insulating barriers, which effectively impede the flow of electric current.

The software used for numerical simulations, Simcenter Star CCM+, requires certain parameters as boundary conditions to ensure the equations can be solved accurately without leading to an overconstrained system. This avoids inconsistencies and secures a unique solution. Some parameters are unknown or have not been documented in the literature. To circumvent this issue, we resorted to mathematical estimations to deduce these parameters from available experimental data. This involved calculating the electric potential and surface resistance for the regions with hair cells and the electric current and electric surface resistance for the regions with dark cells. We have consolidated these calculated values, along with the data used for estimations, in Supplementary Data 1 for reference.

We determined the hair cell density in each region, denoted as ρ^h by using the number of hair cells per area as found in human studies[1]. This reference provided a correlation between hair cell density and subject age. Consequently, the age of the membranous labyrinth anatomy is assumed to correspond to the age selected for the boundary conditions determining the electrical currents. By applying the linear regression's slope and intercept from that study, we calculated the specific hair cell density for both cristae ampullares and the utricular macula, targeting an age of 55 years. This age aligns with the average for adults in the USA and EU.

The surface area of each hair cell region, referred to as A^h, and dark cell region, referred to as A^d, were defined manually in the Simcenter Star CCM+ software. We ensured that the resulting surface areas matched those documented in human[2] and animal studies[3].

To calculate the total number of hair cells within each region (n^h), we multiplied the area of the region by the density of hair cells:

$$n^h = \rho^h A^h \tag{S1}$$

Then, we estimated the total electric current for each hair cell region (I^h) by multiplying the number of hair cells by the electric current produced by a single hair cell given as -100 pA from Roberts et al.[4].

$$I^h = n^h i \tag{S2}$$

The negative sign of these values indicates that the current is exiting the membranous labyrinth, moving outward in a direction perpendicular to the surface. The aggregate of these currents gives the total outflow of electric current from the membranous labyrinth (-4.47 μA). Considering the principle of conservation of electric currents within the membranous labyrinth, we assigned an equivalent total current with the opposite sign for dark cell regions, which implies an inflow of current.

Subsequently, we computed the total electric current density in the dark cell regions (J^d) by summing all the electric currents from the hair cell regions and dividing by the total area of these regions:

$$J^d = \Sigma\, I^h / \Sigma\, A^h \tag{S3}$$

With the total current density (stated as $J^d = 0.22\ A/m^2$), we can then determine the electric current for each dark cell region (I^d) by multiplying the current density by the area of the dark cell region.

$$I^d = J^d A^d \tag{S4}$$

This calculated current is assumed to be evenly distributed across the surface of the dark cell regions and is used as a boundary condition in our simulations. Lastly, the surface resistance for both hair and dark cell regions was derived using Ohm's Law, incorporating the electric potential for endolymphatic fluid (set at $V = 0.01\ V$ from[5], as part of the calculation:

$$R^h = I^h A^h / V \qquad \text{(S5)}$$

$$R^d = I^d A^d / V \qquad \text{(S6)}$$

Supplementary Method 2
Model robustness

We tested various boundary conditions to understand their impact on the variables responsible for magnetic vestibular stimulation (MVS) and to assess the robustness of the model. Three parameters we studied were age (modifying hair cell count as a proxy for age[1]), electric potential (V), and electrical conductivity (σ). We evaluated how these parameters influenced the slow-phase velocity (SPV) of horizontal and torsional eye components computed from the shear strain XY stimulus. The vertical component remained null, consistently with our observations in the supine position, as depicted in Fig. 4. Additionally, we assessed how these parameters affected the average electric current density ($\bar{J}$), the average Lorentz force ($\bar{f_l}$), and the maximum endolymph velocity (v_{max}) in the utricle plane shown in Fig. 1c. The results are presented in Supplementary Table S1, expressing the percentage relative variation for each tested parameter compared to the standard conditions chosen for the model: 55 years old, with 0.01 V of electric potential, and conductivities of 1.67 S/m for both endolymph and cupula.

Firstly, the model was tested for different ages ranging from 20 to 90 years. It was observed that all variables, including the resulting SPV, decreased by 0.4% per year (see Supplementary Fig. S1a). This means that young adults aged 20 are expected to have approximately a 13% stronger SPV relative to 55-year-olds, while elderly adults aged 90 have a 13% reduced SPV relative to 55-year-olds. This parallel change indicates that the spatial distribution of all the variables remains unaffected, only their magnitude changes. Therefore, we observe that the MVS is reduced proportionally to the total electric currents as determined by age change. It is important to note that age could affect other neurological factors,

potentially modifying the resulting MVS or eye movement responses. However, our model only considers the mechanical stimulation in the crista ampullaris.

Secondly, we varied the electric potential boundary condition for all hair cell regions within the range of 0.001 to 0.1 V. These changes in electric potential did not affect any of the studied variables when varied proportionally. This is because this variation altered the magnitude of the electric potential in the membranous labyrinth but did not change its distribution. Consequently, the electric currents and Lorentz force remained similar, resulting in no difference in the endolymph displacement or crista ampullaris shear strain. When the relative electric potential between the macula and crista changes, electric currents flow from some hair cell regions to others. However, we suspected this does not have a physiological basis, considering the function of hair cells as a sink for electric currents.

Thirdly, we varied the electrical conductivity of both endolymph and cupulae between 0.167 and 16.7 S/m, yielding different results depending on the scenario considered (see Supplementary Fig. S1b). When the conductivities of the endolymph (σ_e) and cupulae (σ_c) were varied equally, there was no change in the variables, similar to the effect observed with varying the electric potential. However, when the relative change between the endolymph and cupulae conductivities was considered, all variables followed a single-exponential growth function defined as $y = Ae^{-x/t} + y_0$, with $A < 0$. This indicates that when the ratio σ_e/σ_c is less than unity, the variables were more significantly affected, as seen in the relative percentages in the $\sigma_e < \sigma_c$ column in Supplementary Table S1. In the case of $\sigma_e > \sigma_c$, there were still changes but they were smaller due to the character of the exponential function.

For other inner ear tissue values[6], the electrical conductivity in the cupulae is expected to be of the same order of magnitude as that in the endolymph, or slightly lower due to the lower concentration of water in the cupulae compared to the endolymphatic fluid. Given this, we are in the range where $\sigma_e > \sigma_c$, and therefore, significant variations in the numerical SPV results are not expected. In the future, it would be beneficial to determine experimentally the electrical conductivity of the vestibular system structures and model these conditions to understand more precisely how they may affect MVS.

The effect of age should affect all cupula proportionally, and the electrical conductivity is not expected to vary within the same subject. Therefore, these are fixed variables independent of the head position relative to the magnetic field (B field). For this reason, we expect that the sinusoidal response obtained in the normalized SPV shown in Fig. 4 can be generalizable to other subjects with different boundary conditions in the membranous labyrinth. In other words, we conclude that our model is suitable for studying human MVS for selected parameters shown in Supplementary Data 1 and could even be applicable in a wider range of conditions.

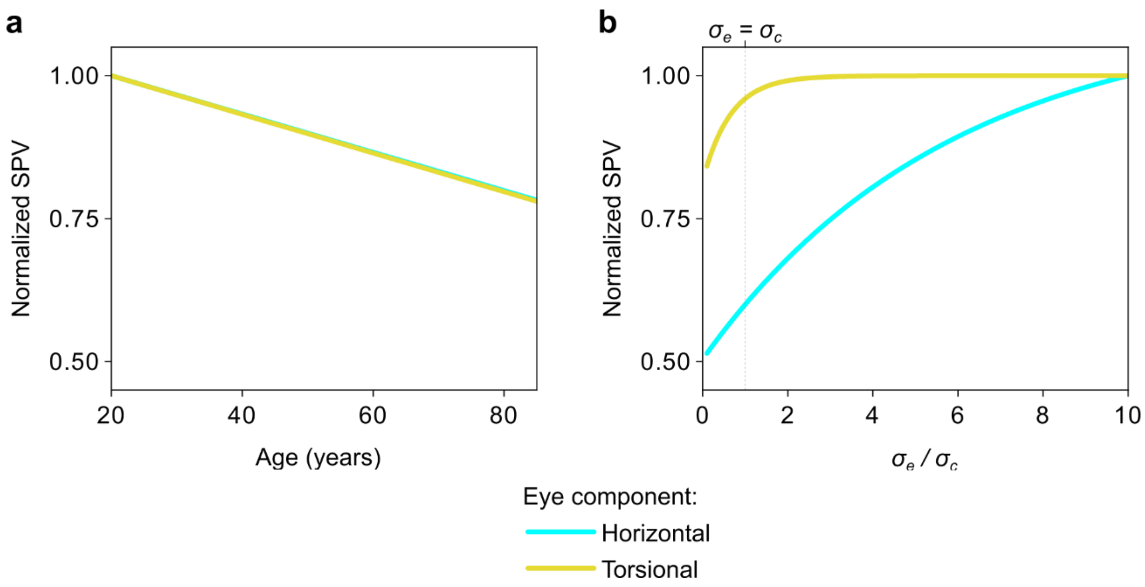

Supplementary Fig. 1 | Effect of boundary conditions on vestibular parameters for determining magnetic vestibular stimulation (MVS). Results are shown for normalized slow-phase velocity (SPV), illustrating (a) the effect of increasing age, represented by hair cell density count, and (b) the relative change in electrical conductivity for both horizontal and torsional components of nystagmus. The vertical component was consistently null in the head position modeled (i.e., supine).

Supplementary Table S1 | Effect of boundary condition changes on variables defining the magnetic vestibular stimulation. The percentage relative variation for different age, electric potential (V), and electrical conductivity (σ) conditions compared to the standard boundary conditions from Supplementary Data 1. The average electric current density ($\bar{J}$), the average Lorentz force ($\bar{f_l}$), and the maximum endolymph velocity (v_{max}) were measured in the section plane of the utricle showed in Fig. 1c. Conductivity results are divided depending on the relation between the electrical conductivity of the endolymph (σ_e) and the cupula (σ_c).

**Variable (% per)**	**ΔAge (year)**	**ΔV (V)**	**$\Delta\sigma$ when $\sigma_e = \sigma_c$ (S/m)**	**$\Delta\sigma$ when $\sigma_e < \sigma_c$ ($\sigma_e / \sigma_c = k$ times)**	**$\Delta\sigma$ when $\sigma_e > \sigma_c$ ($\sigma_e / \sigma_c = k$ times)**
$\Delta\bar{J}$	-0.4	0.0	0.0	2.3	1.0
$\Delta\bar{f_l}$	-0.4	0.0	0.0	2.0	0.9
Δv_{max}	-0.4	0.0	0.0	1.4	0.7
ΔSPV_{hor}	-0.4	0.0	0.0	17.1	5.6
ΔSPV_{ver}	-	-	-	-	-
ΔSPV_{tor}	-0.4	0.0	0.0	14.6	0.4

Supplementary references

1. Merchant, S. N. *et al.* Temporal bone studies of the human peripheral vestibular system. Normative vestibular hair cell data. *Ann Otol Rhinol Laryngol Suppl* **181**, 3–13 (2000).
2. Watanuki, K. & Schuknecht, H. F. A morphological study of human vestibular sensory epithelia. *Arch Otolaryngol* **102**, 853–858 (1976).
3. Kimura, R. S. Distribution, structure, and function of dark cells in the vestibular labyrinth. *Ann Otol Rhinol Laryngol* **78**, 542–561 (1969).
4. Roberts, D. C. *et al.* MRI magnetic field stimulates rotational sensors of the brain. *Curr Biol* **21**, 1635–1640 (2011).
5. Rabbitt, R. D. Semicircular canal biomechanics in health and disease. *Journal of Neurophysiology* **121**, 732–755 (2019).
6. Finley, C. C., Wilson, B. S. & White, M. W. Models of Neural Responsiveness to Electrical Stimulation. in *Cochlear Implants: Models of the Electrically Stimulated Ear* (eds. Miller, J. M. & Spelman, F. A.) 55–96 (Springer, New York, NY, 1990). doi:10.1007/978-1-4612-3256-8_5.
